# Acute COPD exacerbations and in-hospital treatment-related problems: An observational study

**DOI:** 10.1371/journal.pone.0305011

**Published:** 2024-06-06

**Authors:** Oriana Awwad, Rana Abu Farha, Anood Altaharwah, Sarah Sharaya, Abdallah Y. Naser, Haya Tabaza

**Affiliations:** 1 Department of Biopharmaceutics and Clinical Pharmacy, School of Pharmacy, The University of Jordan, Amman, Jordan; 2 Faculty of Pharmacy, Department of Clinical Pharmacy and Therapeutics, Applied Science Private University, Amman, Jordan; 3 Faculty of Pharmacy, Department of Applied Pharmaceutical Sciences and Clinical Pharmacy, Isra University, Amman, Jordan; Jibjibe Rural Hospital, NEPAL

## Abstract

**Background:**

Treatment-related problems (TRPs) interfere with the ability to attain the desired goals of treatment, adding cost to healthcare systems. Patients hospitalized with acute conditions are at particular risk to experience TRPs. Data investigating such burden in patients with acute exacerbation of COPD (AECOPD) is generally scarce with no studies ever conducted in Jordan. This study aimed to investigate and categorize TRPs among patients hospitalized with AECOPD in Jordan, and to estimate their cost savings and cost avoidance.

**Methods:**

This was a retrospective population-based cohort study. Patients’ cases of AECOPD admitted to the study site from Jan 2017 to Jul 2021 were identified from the electronic clinical database and screened for eligibility. TRPs were identified/categorized using AbuRuz tool and assessed for their severity. Cost saving was estimated by calculating all the extra costs. Cost avoidance was estimated according to Nesbit method.

**Results:**

A total of 1243 (mean±SD 3.1±1.5) and 503 (mean±SD 1.3±1.2) TRPs were identified during hospitalization and at discharge respectively, of which 49.4% and 66.7% were classified as “unnecessary drug therapy”. In 54.5% of the cases, systemic corticosteroid was administered for a period longer than recommended. Most of the TRPs were of moderate severity. The total direct cost saving, and cost avoidance were estimated to be 15,745.7 USD and 340,455.5 USD respectively.

**Conclusion:**

The prevalence and cost of TRPs among AECOPD patients is a concern requiring attention. The study results implicate integrating interventions such as embracing clinical pharmacists’ role in the respiratory care units to optimize patients’ management.

## Introduction

The ultimate goal of any disease treatment plan is to achieve the optimum therapeutic outcomes and improve patients’ quality of life avoiding additional harm. Although pharmacological therapies are considered crucial for the management of diseases, these might be associated with treatment-related problems (TRPs) [1–3]. These are known as events or circumstance associated with the patient management that can actually or possibly impede the ability to attain the defined goals of treatment [4]. Unresolved TRPs can increase morbidity and mortality, reduce the patients’ quality of life, increase emergency department and outpatient clinic visits, prolong the length of hospital stay and increase the economic burden on healthcare systems [5–8].

Conditions requiring hospitalization have been showed to be particularly critical for the provision of appropriate patient care since TRPs are relatively common during these conditions, causing burden to patients and healthcare systems [7,9,10]. Chronic obstructive pulmonary disease (COPD) is a chronic lung condition of persistent airflow limitation commonly associated with acute exacerbations. AECOPD, or acute exacerbation of COPD, is characterized by worsening of respiratory symptoms necessitating additional management and sometimes patient’s hospitalization [11]. Despite TRPs have been widely investigated [5], evidences remain scarce among patients with COPD, particularly during exacerbation requiring hospital admission [6,12,13]. Among the middle east and north Africa region, Jordan had the second-highest COPD prevalence with a rate of 5.4% [14]. Despite this, there is lack of clinical research among these patients in general, with no studies ever investigating the burden of AECOPD in particular. This study aimed to assess and categorize TRPs among patients’ cases hospitalized with AECOPD in Jordan, and to estimate their potential cost saving and cost avoidance.

## Methods

### Study settings and patients’ cases

This was a retrospective population-based cohort study conducted at the University of Jordan Hospital. The study site is one of the largest tertiary care referral centers in Amman, the capital of Jordan. It receives patients from the central urban, rural, and surrounding areas [15]. All patients’ cases of AECOPD admitted to the study center during Jan 2017 to Jul 2021 were identified from the electronic clinical database using the ICD-10 code (J44. 1) and screened for inclusion.

Adult patients with AECOPD (≥18 years), having a past medical record at the hospital site since at least one year before their hospitalization, were eligible for inclusion in the study. Clinical cases were excluded if: 1) the patient had an incomplete medical file lacking key information for the assessment of TRPs (e.g., patient’s progress note, or medications sheet) or 2) was admitted with another concomitant medical illness also requiring urgent acute care at the hospital site (e.g., acute kidney injury, and diabetic ketoacidosis).

### Ethical approval

The study was conducted in accordance with the Declaration of Helsinki and was approved by the Institutional Review Board (IRB) committee of the site hospital (Ref. n. 1012021/3185). Because of the retrospective nature of this study, informed consent was exempted. The patients’ medical files were accessed using the hospital electronic archiving medical system, by the research team, from 8^th^ of February 2021 till October 2021. The IRB approval was obtained on date 8^th^ of February 2021 and remained valid for 12 months. This means the research team was able to access data from 8^th^ of February 2021 to 8^th^ of February 2022, however data collection was completed earlier in October 2021.

### Data collection

A structured data collection form was used to collect the patients’ demographic characteristics (such as age and gender), the characteristics of the chronic and acute clinical conditions (such as exacerbation history, comorbidities, exacerbation severity, medications) and all the in-hospital related costs.

The exacerbation severity was assessed using the Anthonisen criteria which take into consideration the patients’ symptoms as a severity metric [16]. The exacerbation is thus considered mild, moderate, or severe whether the patient presents respectively with one, two or three of the following cardinal symptoms: increased sputum volume, increased sputum purulence, increased dyspnea.

All this information was collected from the patients’ electronic medical files and the respective detailed medical invoices retrieved from the hospital’s accounting system. All data were coded and de-identified.

### Assessment of treatment-related problem

Any pharmacotherapy used to manage the AECOPD, as well as the duration of systemic corticosteroid administration, were assessed for its appropriateness based on the Global Initiative for Chronic Obstructive Lung Disease (GOLD) guideline. Whenever inappropriate the management was considered a TRP. This also included the assessment of stress ulcer prophylaxis since its use was related to the main clinical condition of admission (AECOPD).

Whenever the management was not adequately addressed in the GOLD guideline, the UpToDate [17] was used as second reference. This applied also to the evaluation of the need for stress ulcer prophylaxis [18]. Furthermore, treatment plan medications were checked for potential category ‘X’ drug-drug interaction (avoid combination) using the Lexicomp® Drug Interactions-Online database [19].

All the identified TRPs were evaluated and classified using a validated [9,20–22] tool published by AbuRuz et al. [23]. This tool assesses TRPs during hospitalization and at discharge. According to the tool, TRPs are classified into five major categories during hospitalization: ‘unnecessary drug therapy’, ‘untreated condition’, ‘efficacy’, ‘safety’ and ‘miscellaneous;’. TRPs at discharge are classified into four major categories: ‘unnecessary drug therapy’, ‘ineffective/incomplete drug therapy’, ‘actual and potential adverse drug reactions’, and ‘actual and potential drug interactions’. Ultimately, each of the major classes of TRPs during hospitalization is further categorized into minor subclasses [23].

### Severity of treatment-related problem

After their identification, the severity of TRPs was assessed using Nesbit’s method [24] based on their risk to cause patient’s harm. According to Nesbit et al. [24] TRPs severity can be categorized into 1) No harm: no potential adverse drug event (ADE) (hence no clinical influence) 2) Very Mild: potential ADE with minimal and marginal clinical influence 3) Mild: potential ADE with significant clinical influence 4) Moderate: potential ADE with serious clinical influence or 5) Severe: potential ADE very likely to cause severe lasting reaction or death.

### Economic evaluation

For the economic evaluation, both the direct cost saving, and cost avoidance were assessed for each TRP, from provider perspective.

Cost saving was the extra cost of medications (inpatient medications, outpatient medications, corticosteroids) and medical procedures/supplies identified as TRPs. This was estimated directly by summing all the costs judged unnecessary (extra) to the treatment plan.

Cost avoidance was the cost that could have been avoided in the presence of interventions preventing TRPs and their potential ADE’s. Based on Nesbit et al. method [24] each TRP can be assessed for its risk (probability) to cause an ADE. Accordingly, a panel of three clinical pharmacists attributed to each TRP a probability risk score from 0.00 (zero) to 0.6 (high) (see S1 Table in S1 File). Cohen’s kappa test was used to examine the inter-rater reliability between the pharmacists’ panel. The probability scores for all TRPs (P) were then combined to generate a total number of preventable ADEs (N).

Based on previous studies, each preventable ADE is assumed to result in two extra days (D) of hospitalization [25–29]. On these bases, and according to Nesbit method, the estimated cost avoidance was calculated using the formula (N)×(D)×(C) where C was the cost of one-day hospital stay (mean inpatient cost per day among the study sample).

Finally, the total cost saving, and cost avoidance were summed up to calculate the total estimated cost of TRPs. Costs are presented in United States dollars at a rate of USD 1 = JOD 0.71.

### Data analysis

Data were coded, entered, and analyzed using Statistical Package for the Social Sciences software (SPSS version 23). Descriptive analyses were performed on patients’ cases demographics, acute and chronic clinical data, TRPs, their categories, costs, and severity. Continuous variables were expressed as mean±SD. Nominal and ordinal variables were presented as frequencies (percentages).

## Results

### Demographic and clinical characteristics of the study cases

A total of 402 cases were included in the study. The mean age of the patients was 67.3 ± 10.8 years, with the majority being males (n = 344, 85.6%), the most reported not to having any allergy (n = 373, 92.8%). Patients had a mean of 2.2 ± 1.0 COPD medications taken prior to admission and a mean number of comorbidities of 2.6 ± 1.7. The majority of the patients presented with symptoms of severe exacerbation (44.8%). The mean number of COPD in-hospital medications was 6.0 ± 1.7. For more details on patients’ demographics and clinical information refer to Table 1.

**Table 1:** Demographic and clinical characteristics of the study cases (n = 402).

Parameter	
**Age (years)** ^**b**^	67.3 (10.8)
**Gender**^**a**^ • Male • Female	344 (85.6) 58 (14.4)
**Allergies**^**a**^ • No • Yes	373 (92.8) 29 (7.2)
**Long term oxygen therapy**^**a**^ • No • Yes	244 (61.2) 156 (38.8)
**Number of comorbidities** ^**b**^	2.6±1.7
**Number of COPD PTA medications** ^**b**^	2.2±1.0
**Number of COPD in-hospital medications** ^**b**^	6.0±1.7
**Severity of COPD exacerbation**^**b**^ • Mild • Moderate • Severe	93 (23.1) 129 (32.1) 180 (44.8)

^a^ Data expressed as n (%).

^b^ Data expressed as mean±SD.

### Treatment-related problems during hospitalization

A total number of 1243 TRPs were identified among the study cases. The three most common TRP classes were “unnecessary drug therapy” (n = 615, 49.4%), “safety" (n = 499, 40.1%) and “efficacy" (n = 68, 5.5%).

**Fig 1:**
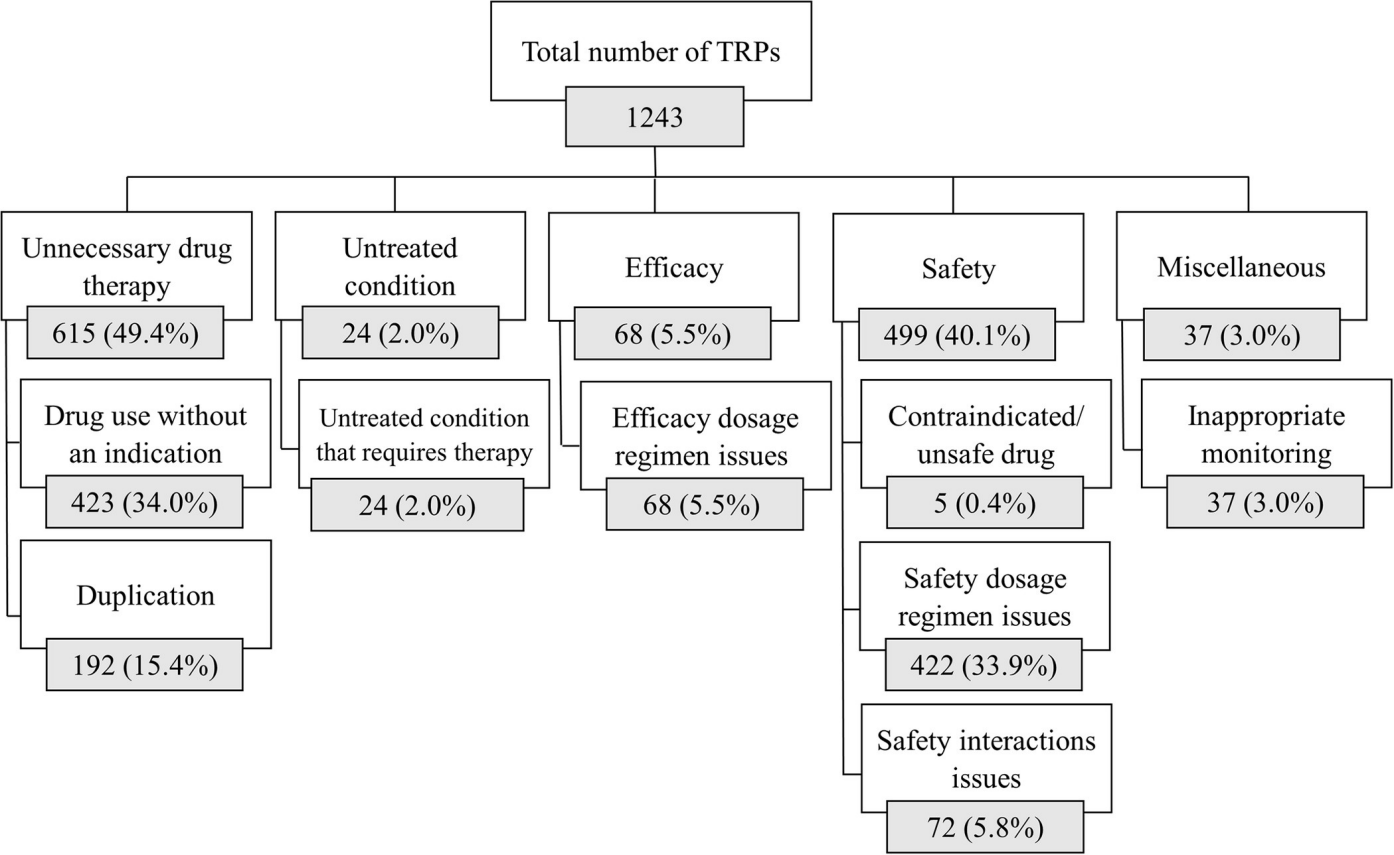
Classification of the TRPs identified among COPD patients during hospitalization (n = 1243). Data is presented as N (%); percentage was calculated from the total number of TRPs.

The most identified medications among “unnecessary drug therapy” TRPs were proton pump inhibitors (n = 222). Corticosteroids were the most associated with “safety” TRPs (n = 405) and “efficacy” TRPs (n = 33). For more details about the classes and sub-classes of TRPs during hospitalization refer to Fig 1, while refer to S2 Table in S1 File for examples on the different types of TRPs.

**Fig 2:**
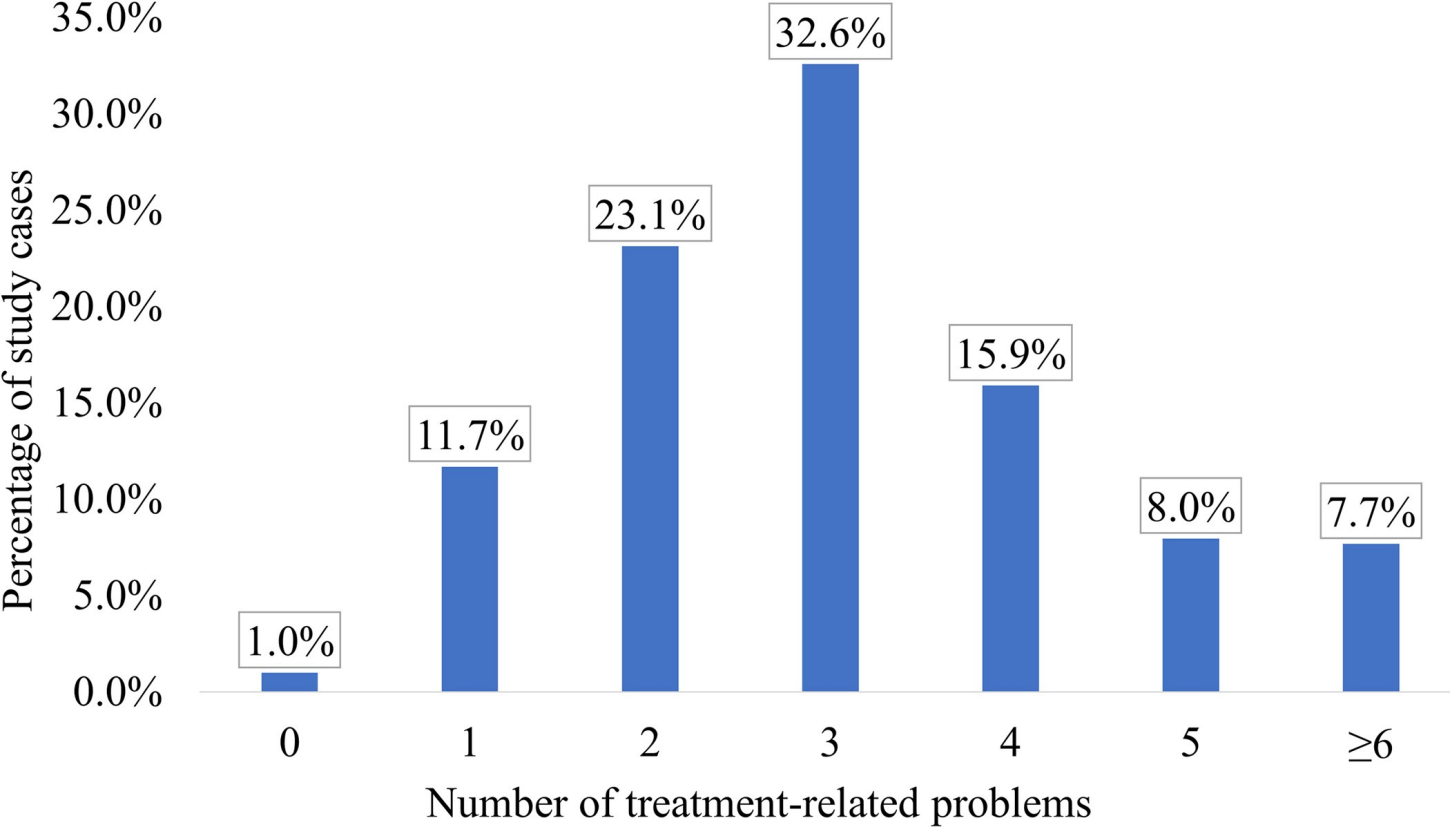
Distribution of TRPs during hospitalization among the study cases (n = 402).

According to the study results, 99% of the cases were found to have at least one TRP during hospitalization, with three TRPs identified in around one third of the cases (32.6%) (Fig 2).

**Fig 3:**
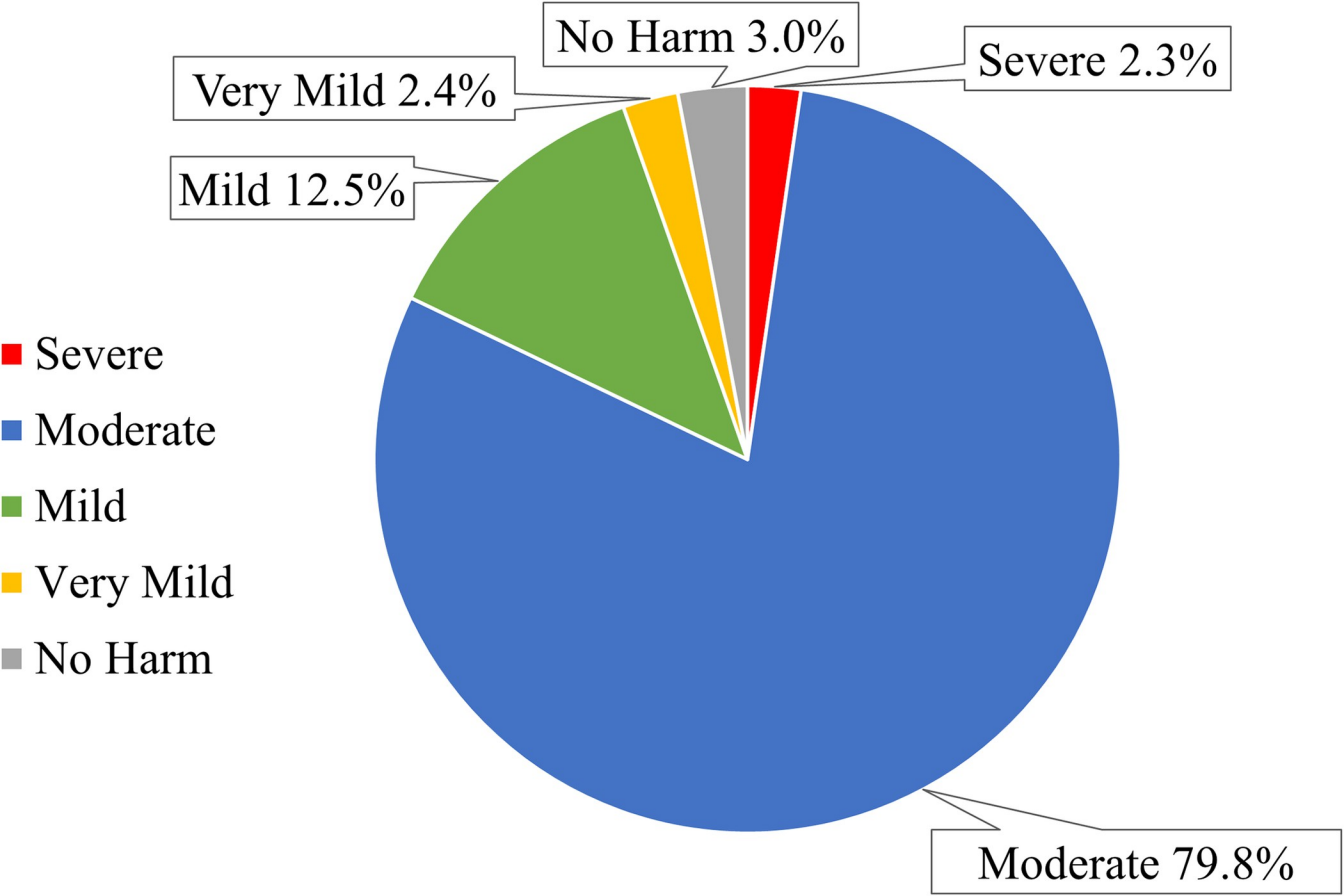
Classification of the TRPs during hospitalization based on their severity. Data is expressed as percentages (n = 1243).

Severity of TRPs during hospitalization ranged from no harm to severe with the most found to be of moderate severity (n = 992, 79.8%) (Fig 3). Examples of the TRPs of different seriousness are presented in S3 Table in S1 File.

### Treatment-related problems at discharge

**Fig 4:**
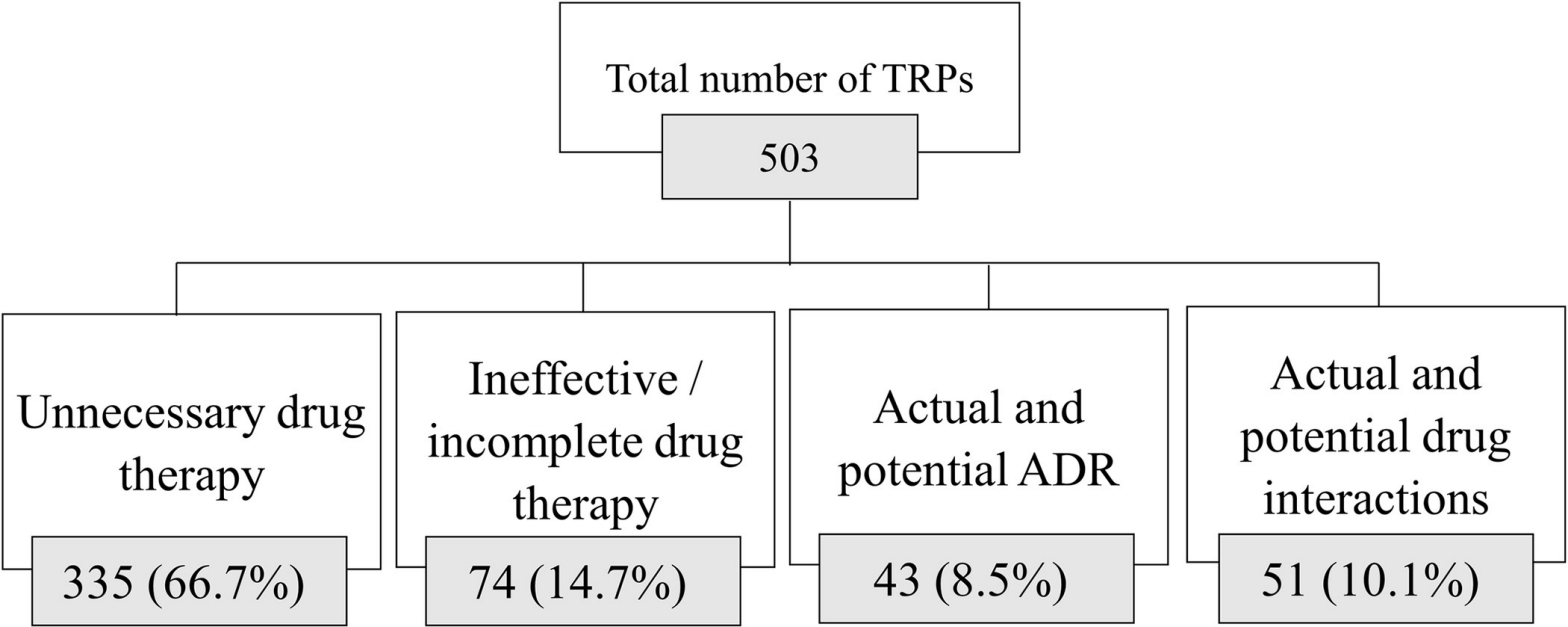
Classification of the TRPs identified among COPD patients at discharge (n = 503). Data is presented as N (%); percentage was calculated from the total number of TRPs.

**Fig 5:**
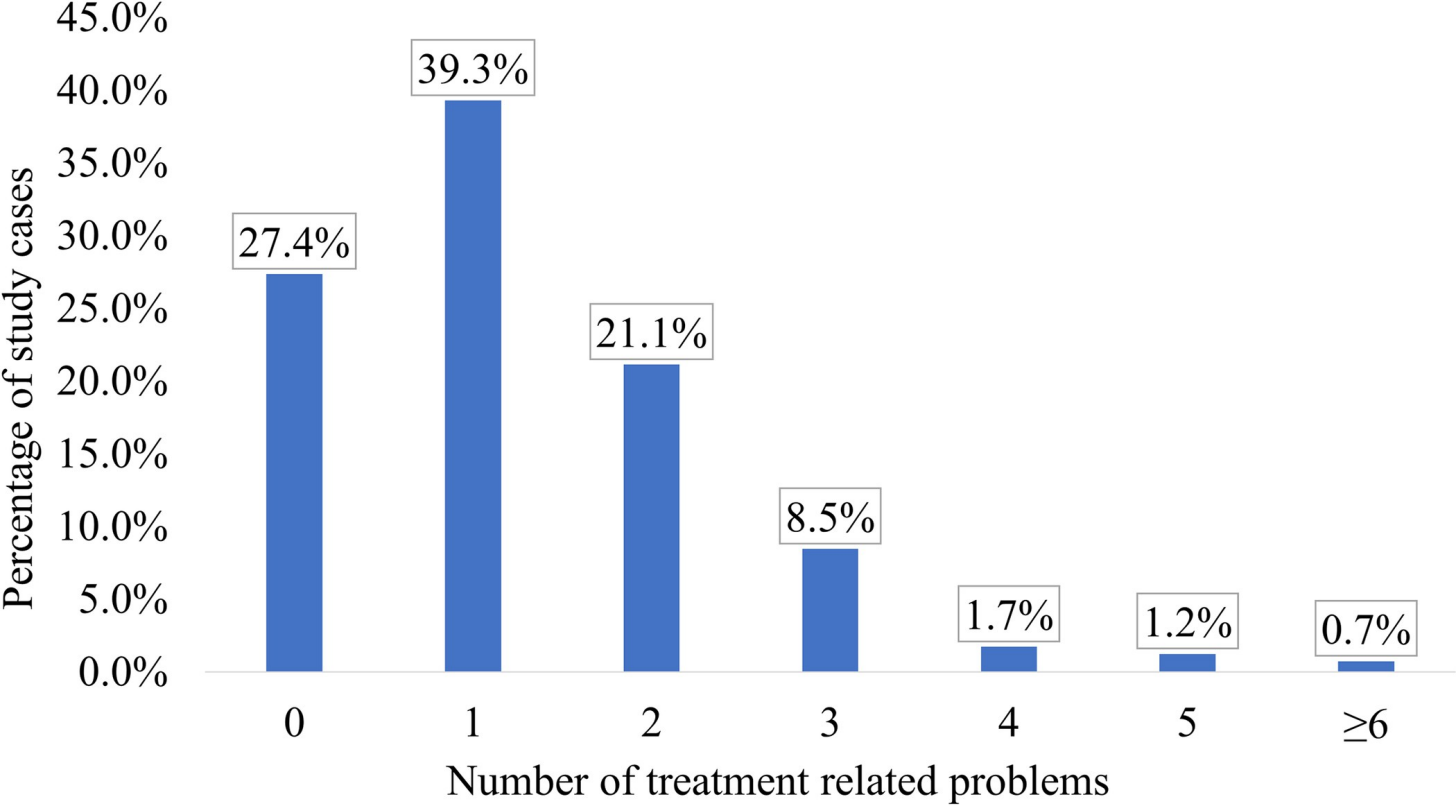
Distribution of TRPs during hospitalization among the study cases (n = 402).

A total of 503 TRPs were identified at discharge among the study cases. Two-third of them were related to unnecessary drug therapy (n = 335, 66.7%), 14.7% (n = 74) to ineffective/incomplete drug therapy, 10.1% (n = 51) to actual and potential drug interaction, and 8.5% (n = 43) were related to actual and potential adverse drug reactions (Fig 4). Most of the study cases (72.6%) showed at least one TRP (Fig 5). Examples of the different types of TRPs at discharge are presented in the S4 Table in S1 File.

**Fig 6:**
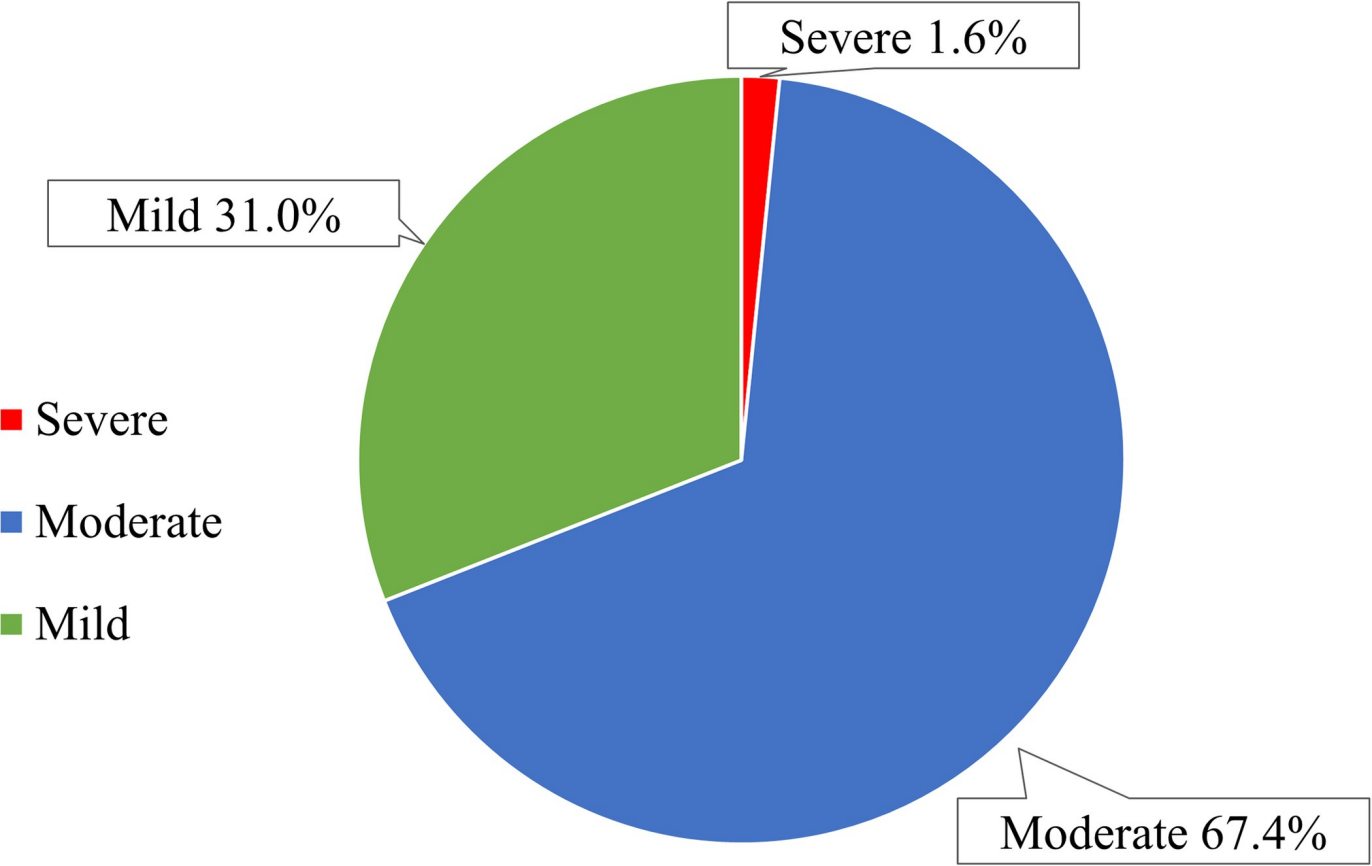
Classification of the TRPs during hospitalization based on their severity. Data is expressed as percentages (n = 503).

In regard to the TRP severity (Fig 6), only 1.6% (n = 8) of them were classified as severe TRPs, the remaining were either moderate (n = 339, 67.4%) or mild (n = 156, 31.0%). Examples of the TRPs of different seriousness are presented in the supporting information (S5 Table in S1 File).

### Systemic corticosteroids

Upon evaluation of the total duration of systemic corticosteroids administration, data revealed that 219 cases (54.5%) were prescribed corticosteroid for a period longer than recommended.

### Cost estimation of COPD TRPs

The TRPs identified among the study cases during hospitalization and at discharge in addition to the longer systemic corticosteroids treatment (n = 1965) resulted in a total extra cost of USD 15,745.7 (USD 7452.6, USD 3559.1, USD 3907.2, and USD 826.8 for inpatient medications, outpatient medications, systemic corticosteroids, and medical procedures/supplies respectively) (Table 2). In addition, when summing all the TRPs’ probabilities scores, the total number of preventable ADEs (N) resulted to be 607.1. Given the mean±SD cost of one day of hospitalization among the study cases was found to be USD 280.39±109.45 the total cost avoidance was thus estimated to be USD 340,455.5. The total sum of cost savings and cost avoidance was USD 356,201.2 (Table 2).

**Table 2:** Estimated cost of total identified TRPs (n = 1965).

Cost savings	Cost avoidance	Total
INPATIENT MEDICATIONS: 7452.6 USD	Total number of preventable ADEs: 607.1	
OUTPATIENT MEDICATIONS: 3559.1 USD	Mean inpatient cost of one day of hospitalization: 280.39 USD	
SYSTEMIC CORTICOSTEROIDS: 3907.2 USD		
MEDICAL PROCEDURES & SUPPLIES: 826.8 USD		
Total cost savings: 15,745.7 USD	**Total cost avoidance:** **340,455.5 USD**	**Total estimated cost:** **356,201.2 USD**

## Discussion

This is one of the few studies investigating TRPs among patients admitted with AECOPD worldwide and the first in Jordan [6,13]. A high prevalence of TRPs were identified, the most were of moderate severity. Almost all the study cases experienced at least one TRP during hospitalization with a mean±SD of 3.1±1.5 per case. This incidence is high compared to Li et al. (2019) study reporting a mean of 1.6 drug-related problems per patient among only 56.7% of the study population [13] which might be due to differences in the study design and the tool used to assess TRPs, however, it also reflects poor clinical knowledge and practices in the management of AECOPD in Jordan. Reports from Jordan also showed a high prevalence of TRP in hospitalized internal medicine patients, general chronic disease outpatients, and hemodialysis patients [9,30,31].

Unnecessary drug therapy and safety issues were the most common major class of TRPs representing around 90% of all the TRPs during hospitalization. Drug use without indication and safety dosage regimen issues were the respectively most common subclasses. These results are similar to previous data also reporting most of the TRPs being related to treatment safety, treatment effectiveness and unnecessary drug treatment with most of the problems associated with drug selection, dose selection, and treatment duration [13].

Interestingly, proton pump inhibitors (PPIs) were the most common class of medications used without indication. In regards to safety dosage regimen issues, corticosteroids were commonly administered at doses higher than the recommended ones [11,17]. PPI were previously observed to be misused generally in health practices including Jordan [32,33]. Given the increased risk of pneumonia among COPD patients treated with proton pump inhibitors, interventions are needed to restrict this inappropriate practice [34–37].

When looking at the TRPs at discharge, the study results showed 72.6% of the patients experiencing at least one TRP, with a mean±SD of 1.3±1.2 TRP per patient. Similarly, results published by Apikoglu-Rabus et al., reported an average of 1.6 drug related problem per COPD patient visiting the outpatient clinic [12].

While unnecessary drug therapy was the most common class of TRP, accounting for ~67% of all the TRPs at discharge, previous studies showed treatment effectiveness or adverse drug reactions to be the most common treatment problems [12,38]. In this line, most of the study cases were shown to receive duplication of inhaled corticosteroids. The observed differences might be attributed not only to the research designs and TRP classification tools, but also to variations in the clinical practices adopted among the different study populations. This requires healthcare providers to comply more with the most updated clinical guideline for the treatment of COPD exacerbation as these reports provide the most important evidence in this regard (GOLD).

In addition to the aforementioned treatment problems, most of the study cases also received systemic corticosteroids for a longer duration than the recommend one. Systemic corticosteroids are known to fasten recovery and decrease relapse rate in AECOPD; however, they can also aggravate underlying chronic conditions such as diabetes, hypertension, gastrointestinal ulcers without additional benefits to the COPD exacerbation [11,39–41]. Furthermore, in a recent nationwide study higher risk of venous thromboembolism and all-causes mortality were observed among patients with AECOPD treated with long-course of oral corticosteroids in comparison to short-course [42].

Beside posing a threat to patients’ safety, TRPs are also associated with extra cost on patients and health care systems [5,29,43,44]. In this study, the overall TRPs were associated with an estimated total cost savings and cost avoidance of 15,745.7 USD and 340,455.5 USD respectively.

Clinical pharmacists can play key role in the identification, resolution, and prevention of TRPs effectively contributing also to important healthcare savings [4,29,45–48]. Clinical pharmacist interventions were reported to decrease the incidence of ADEs by 66–78% in different healthcare departments [49–51]. Moreover, healthcare systems involving clinical pharmacists resulted in a shorter hospital stay and lower medication cost, with an average cost savings of 400$ per hospital admission and a benefit-cost ratio of 6.03:1 [52]. In Jordan, Al‐Qudah et al. reported a substantial cost-benefit of TRP-related pharmacist interventions among general outpatients with chronic diseases [30]. Despite this, their presence is still challenging and not fully implemented in the health care systems in Jordan [53].

Changes at the policy and regulation levels are needed to better integrate clinical pharmacists and expand their roles in the respiratory medicine departments in order to optimize patients’ management, assure their safety and reduce costs on healthcare systems. Actions should also be also taken among healthcare professionals to improve drug therapy practices through the provision of training and information updates regarding the most recent clinical management guidelines.

In conclusion, this is one of the few studies investigating the TRPs and their cost among patients with AECOPD. Several TRPs were identified among the study participants representing a concern for patients’ safety and an important impact on healthcare system expenditure. This represents a concern for patients’ safety and healthcare that require attention. TRPs related to ineffective drug therapy and safety were the most common. The study results implicate the implementation of interventions such as embracing the role of clinical pharmacists in the respiratory care units to optimize the patients’ management.

Limitations of the study include its retrospective nature which mandated the exclusion of cases with incomplete data. Patient-related TRPs such as medication knowledge and adherence were also not assessed. In addition, this was a single center study that, however, might pave the way for future prospective studies to be conducted in multiple centers in Jordan.

## Supporting information

S1 File Supplementary methodology and results. https://figshare.com/s/5f0187927a827027ad73.
(DOCX)
