# Supplementary material for: Acute COPD exacerbations and in-hospital treatment-related problems: An observational study
Source: PLoS One. 2024 Jun 6;19(6):e0305011. doi: 10.1371/journal.pone.0305011 (PMC11156384; doi:10.1371/journal.pone.0305011)
Supplement: S1 File — https://figshare.com/s/5f0187927a827027ad73. (DOCX) [file pone.0305011.s001.docx]

**Table S1.** Cost avoidance estimation using Nesbit method (1)

| **Probability of ADE occurrence** | **Probability score** | **Definition and examples** |
| --- | --- | --- |
| No Harm | 0 | No harm is expected. e.g. redundant assessment of arterial blood gases. |
| Very Mild | 0.01 | Minimal harm is expected, potentially with marginal clinical relevance. e.g. unjustified use of oxygen therapy. |
| Mild | 0.1 | Harm is expected, potentially with significant clinical relevance. e.g. the administration of subtherapeutic doses of systemic corticosteroids and LABA/LAMA. |
| Moderate | 0.4 | Harm is expected, potentially with serious clinical relevance. e.g. duplication of inhaled corticosteroids, supratherapeutic dosing of systemic corticosteroids. |
| Severe | 0.6 | Fatal harm is expected, could potentially result in life-threatening consequences. e.g. the absence of venous thromboembolism prophylaxis. |

^LABA = long-acting beta agonist. LAMA = long-acting muscarinic antagonist^

**Table S2.** Examples of treatment-related problems during hospitalization

| Type of TRP | Example |
| --- | --- |
| *Unnecessary drug therapy* | |
| Drug use without an indication | The addition of an antibiotic (such as levofloxacin 750mg) while the patient does not meet the GOLD criteria for antibiotics prescription. |
| Duplication | The use of 2 different systemic corticosteroids such as methylprednisolone and hydrocortisone at the same time, while the patient needs just one agent. |
| *Untreated condition* | |
|  | The patient was not given a venous thromboembolism (VTE) prophylaxis despite its need as part of the management. |
| *Efficacy* | |
| Efficacy dosage regimen issues | A patient was given a tiotropium 2.5 mcg one inhalation once daily, which is lower than the recommended effective dose of 2 inhalations once daily without justification. |
| *Safety* | |
| A current drug is contraindicated/unsafe for patient condition and should be stopped, monitored or replaced | The use of theophylline 300mg once daily during the AECOPD, while its use is contraindicated. |
| Safety dosage regimen issues | A patient was given methylprednisolone 40mg 4 times daily, which exceeded the maximum recommended dose. |
| Safety interactions issues | The concurrent use of loratadine along with ipratropium, as loratadine can enhance the anticholinergic effects of ipratropium. |
| *Miscellaneous* | |
| Inappropriate monitoring | The unjustified assessment of arterial blood gases several times during hospitalization in patients with normal oxygen saturation and not on oxygen therapy. |

**Table S3.** Examples of TRPs severity during hospitalization

| Severity classification | Example |
| --- | --- |
| No harm | The assessment of unnecessary arterial blood gases. |
| Very Mild | The use of unnecessary oxygen treatment. |
| Mild | The use of lower than the recommended dose of systemic corticosteroids. |
| Moderate | The use of higher than the recommended dose of inhaled corticosteroids. |
| Severe | The absence of VTE prophylaxis during hospital admission. |

**Table S4.** Examples of TRPs on discharge medications

| Type of TRP | Example |
| --- | --- |
| *Treatment-related problems on discharge medications* | |
| Unnecessary drug therapy | The addition of an antibiotic (such as levofloxacin 750mg) while the patient does not meet the GOLD criteria for antibiotics prescription. |
| Ineffective/incomplete drug therapy | A patient was discharged without adding LABA or LAMA to the treatment regimen. |
| Actual and potential ADR | A patient was discharged on budesonide 160 mcg/formoterol 4.5 mcg 2 inhalations three times daily which is higher than the maximum allowed dose (maximum 2 inhalations twice daily ). |
| Actual and potential drug interactions | The concurrent use of loratadine along with ipratropium, as loratadine enhance the anticholinergic effects of ipratropium. |

**Table S5.** Examples of TRPs severity at discharge

| Severity classification | Example |
| --- | --- |
| Mild | The addition of unnecessary antihistamine. |
| Moderate | The prescription of unnecessary antibiotic such as levofloxacin 750mg once daily for 7 days. |
| Severe | The drug-drug interaction between loratadine and orphenadrine. |

References:

1. Nesbit TW, Shermock KM, Bobek MB, et al. Implementation and pharmacoeconomic analysis of a clinical staff pharmacist practice model. American journal of health-system pharmacy : AJHP : official journal of the American Society of Health-System Pharmacists. 2001;58(9):784-90. Epub 2001/05/16. doi: 10.1093/ajhp/58.9.784. PubMed PMID: 11351918.
